# 孕期类固醇激素的液相色谱-串联质谱检测方法建立及与化学发光法一致性分析

**DOI:** 10.3724/SP.J.1123.2025.07001

**Published:** 2026-03-08

**Authors:** Weixiang WU, Lihong WU, Fuqiang DIAO, Youwen LUO, Chunming GU, Mingyong LUO

**Affiliations:** 南方科技大学附属妇女儿童医院临床检验中心，广东省妇幼保健院，广东 广州 511443; Department of Clinical Laboratory，Women and Children’s Hospital，Southern University of Science and Technology，Guangdong Women and Children Hospital，Guangzhou 511443，China

**Keywords:** 液相色谱-串联质谱法, 化学发光免疫分析法, 类固醇激素, 一致性分析, 睾酮, liquid chromatography-tandem mass spectrometry （LC-MS/MS）, chemiluminescence immunoassay, steroid hormone, consistency analysis, testosterone

## Abstract

类固醇激素在妊娠和胎儿发育中发挥重要作用，其准确检测对评估孕期内分泌状态具有重要临床价值。当前我国临床常用的化学发光免疫分析法（CLIA）在方法学上存在一定局限性，而液相色谱-串联质谱法（LC-MS/MS）虽已被国际公认为更可靠的检测手段，但在我国尚未得到广泛应用，且针对孕妇群体中两种检测方法的一致性评价仍缺乏系统性研究。本研究建立并验证了一种基于LC-MS/MS的多激素检测方法，可同时测定血清中皮质醇、硫酸脱氢表雄酮（DHEAS）、睾酮和17*α*-羟孕酮（17-OHP）。该方法线性决定系数>0.999，定量限（LOQ）为0.008~0.137 ng/mL，加标回收率为92.1%~110.9%，相对标准偏差（RSD）为3.2%~9.0%，可满足临床检测要求。在94例孕妇样本中的一致性分析结果显示，两种方法测得结果在所有激素水平上均存在显著差异（*P*均<0.001），其中LC-MS/MS测得的皮质醇和DHEAS水平显著低于CLIA，而睾酮和17-OHP水平则显著高于CLIA。Bland-Altman分析显示，皮质醇（-56.29 ng/mL）和DHEAS（-1 079.68 ng/mL）存在负向偏倚，睾酮（0.22 ng/mL）和17-OHP（0.89 ng/mL）存在正向偏倚，且偏倚随浓度升高而扩大。两种方法在4种激素检测中均具有高度正相关（*P*<0.001，*r*=0.819~0.974）。Passing-Bablok回归分析结果发现，皮质醇的一致性回归方程为*C*_LC-MS/MS_=-3.58+0.57*C*_CLIA_，存在明显的比例性偏倚；DHEAS的方程为*C*_LC-MS/MS_=-59.77+0.38*C*_CLIA_，存在固定性偏倚和比例性偏倚；睾酮的方程为*C*_LC-MS/MS_=-0.05+1.30*C*_CLIA_，存在固定性和比例性偏倚，且随着浓度升高差异逐渐加大；17-OHP的方程为*C*_LC-MS/MS_=0.10+1.75*C*_CLIA_，存在比例性偏倚，且差异随浓度增加而扩大。本研究不仅建立了高灵敏度、高准确性的多激素LC-MS/MS检测方法，也揭示了其与CLIA之间的系统性差异。研究结果提示临床医生在解读检测结果时，需充分考虑检测方法的差异性，降低因检测误差导致的临床误诊风险。未来应在更大样本中验证其临床适用性，并推动LC-MS/MS在孕期内分泌监测中的标准化应用。

## Abstract (en)

Steroid hormones play a critical role in maintaining pregnancy and supporting fetal development. Accurate quantification of these hormones is essential for evaluating the endocrine status during pregnancy. While chemiluminescence immunoassay （CLIA） is widely used in clinical practice in China， it has inherent methodological limitations. In contrast， liquid chromatography-tandem mass spectrometry （LC-MS/MS） has been internationally recognized for its superior analytical performance but remains underutilized in Chinese obstetric practice. Moreover， systematic evaluations comparing these two methods in pregnant women are still lacking. This study aimed to develop and validate an isotope-dilution LC-MS/MS method for the simultaneous quantification of four key steroid hormones， including cortisol， dehydroepiandrosterone sulfate （DHEAS）， testosterone， and 17-hydroxyprogesterone （17-OHP）. In addition， a consistency analysis was conducted between LC-MS/MS and CLIA among pregnant women. Method validation followed international bioanalytical guidelines， evaluating linearity， limits of quantification （LOQs）， recovery， and precision. Serum samples from 94 pregnant women were collected at Guangdong Women and Children Hospital between January 2022 and December 2023， and were analyzed using both LC-MS/MS and CLIA. Method comparison was performed using paired *t*-tests， Pearson correlation， Bland-Altman plots， and Passing-Bablok regression to assess concentration differences， correlation， and systematic biases between the two methods. In this study， the established LC-MS/MS method exhibited excellent performance， with linear coefficients of determination >0.999， LOQs of 0.008-0.137 ng/mL， spiked recoveries of 92.1%-110.9%， and intra-assay relative standard deviations （RSDs） of 3.2%-9.0%， meeting the requirements for clinical detection. In a cohort of 94 pregnant women， paired *t*-tests revealed significant differences in hormone concentrations measured by LC-MS/MS and CLIA （all *P<*0.001）. Specifically， LC-MS/MS yielded significantly lower values for cortisol （64.31 vs. 120.60 ng/mL） and DHEAS （532.44 vs. 1 612.1 ng/mL）， but higher values for testosterone （1.10 vs. 0.88 ng/mL） and 17-OHP （2.00 vs. 1.07 ng/mL）. Bland-Altman analysis further revealed obvious negative proportional biases for cortisol and DHEAS， with bias values of -56.29 ng/mL and -1 079.68 ng/mL， respectively. Additionally， the biases increased with the increase of concentration. In contrast， testosterone and 17-OHP showed positive biases， with bias values of 0.22 ng/mL and 0.89 ng/mL， respectively. The two methods showed extremely strong positive correlations in the detection of the four hormones （*P*<0.001， *r*=0.819-0.974）， among which the correlation of testosterone detection results was the highest （*r*=0.974）. In the Passing-Bablok regression analysis， the consistency regression equation for cortisol was *C*_LC-MS/MS_=-3.58+0.57*C*_CLIA_. The confidence interval （CI） of the slope did not include 1， indicating obvious proportional bias. The consistency regression equation for DHEAS was *C*_LC-MS/MS_=-59.77+0.38*C*_CLIA_. The CIs of the intercept and slope did not include 0 and 1， respectively， suggesting the existence of fixed and proportional biases. The consistency regression equation for testosterone was *C*_LC-MS/MS_=-0.05+1.30*C*_CLIA_. The CIs of the intercept and slope did not include 0 and 1， respectively， indicating the existence of fixed and proportional biases. In addition， the differences increased with the increase of concentration. For 17-OHP， the regression equation was *C*_LC-MS/MS_=0.10+1.75*C*_CLIA_. Though the intercept was not statistically significant， the slope deviated significantly from 1， indicating a proportional bias. Additionally， LC-MS/MS values were significantly higher， and the difference increased with rising concentration. In conclusion， the established LC-MS/MS method offers high sensitivity， precision， and accuracy for the simultaneous quantification of multiple steroid hormones. The method reliably measured cortisol， DHEAS， testosterone， and 17-OHP levels in maternal serum. Additionally， our results revealed statistically significant differences between the two methods across all four hormones in pregnant women， with biases varying by concentration. These findings highlight the importance for clinicians to consider methodological differences when interpreting hormone test results during pregnancy， in order to improve the accuracy of endocrine assessment and reduce the risk of misdiagnosis due to analytical discrepancies. Future studies should incorporate large-scale maternal cohorts covering all stages of pregnancy and high-risk populations to further assess the clinical applicability of different methods and promote the standardization of LC-MS/MS for obstetric endocrine monitoring. In parallel， trimester-specific reference intervals should be established to support clinical interpretation and risk evaluation.

类固醇激素由胆固醇合成，在妊娠期显著升高，参与胎盘发育、免疫调节和胎儿生长的全过程^［1］^。研究表明，母体睾酮水平升高会增加新生儿低出生体重和早产风险^［2］^。此外，孕期高水平的皮质醇和硫酸脱氢表雄酮（dehydroepiandrosterone sulfate，DHEAS）与不良妊娠结局存在显著关联^［3，4］^，而17*α*-羟孕酮（17*α*-hydroxyprogesterone，17-OHP）水平对胎儿出生体重呈现出性别特异性^［5］^。准确评估孕期激素水平对于评估胎儿发育状况、诊断妊娠相关并发症以及深入理解孕期内分泌变化具有重要意义。然而，由于孕期激素浓度波动明显、种类繁多、部分关键激素（如睾酮、17-OHP）含量极低但生物学意义重大，因此对检测方法的准确性和稳定性提出了更高要求^［6］^。

目前，临床常用的类固醇激素检测方法主要包括化学发光免疫分析（chemiluminescence immunoassay，CLIA）和液相色谱-串联质谱（liquid chromatography-tandem mass spectrometry，LC-MS/MS）。其中，LC-MS/MS被国际广泛认为是类固醇激素测定的金标准方法，具备高特异性和高灵敏度，能同时对多种激素进行准确定量^［7］^。然而，在我国当前临床实践中，CLIA因其自动化程度高、操作简便，适用于大规模筛查而在临床中广泛应用。已有研究表明，CLIA在检测结构相似的类固醇激素时，容易受到交叉反应、基质效应干扰及灵敏度和特异性不足等因素的影响，尤其在目标激素浓度较低或个体生理差异较大时，可能产生系统性偏倚。在孕妇群体中，妊娠期间伴随血浆稀释、激素结合蛋白水平波动及肝肾代谢能力的改变，这些生理变化可能进一步削弱CLIA的检测准确性^［6］^。尽管已有研究在儿童或成人群体中对CLIA与LC-MS/MS进行了比较，但针对妊娠人群的相关研究仍较为缺乏^［8，9］^。

准确获取孕期激素水平不仅对科学研究至关重要，在临床实践中也具有重要意义。不同检测方法可能导致激素水平的显著差异，进而影响对妊娠结局的风险评估和干预决策。因此，深入比较CLIA与LC-MS/MS在妊娠人群激素检测中的一致性，对于提升孕期内分泌监测的准确性和临床干预的科学性具有重要价值。本研究以妊娠早期样本为基础，选取临床常见的4种类固醇激素（皮质醇、DHEAS、睾酮及17-OHP），系统比较CLIA与LC-MS/MS两种检测方法的结果，并通过多种统计学方法评估二者的一致性差异，旨在为孕期激素检测方法的优化及临床应用提供循证依据。

## 1 实验部分

### 1.1 人群资料

本研究一致性分析所用样本来自2022年1月至2023年12月期间在广东省妇幼保健院接受产前检查并符合纳入标准的孕妇，由本院出生队列中随机抽取。纳入标准如下：（1）自然受孕；（2）胎龄<16周；（3）年龄≥16岁；（4）无恶性肿瘤、自身免疫病、心脏病、传染病或内分泌疾病。排除标准如下：（1）缺乏规律产检；（2）未在本院分娩；（3）多胎妊娠、死产、流产或畸形；（4）采样前1个月内接受激素治疗。最终纳入94例孕妇用于一致性分析，纳入样本与总体在年龄和孕前体重指数（body mass index，BMI）上无显著差异。因部分样本血清量不足，17-OHP的一致性分析最终纳入了72对样本。该研究获得了广东省妇幼保健院医学伦理委员会的批准（No. 202201072）。

### 1.2 样本采集和数据提取

在取得知情同意后，由专业医师于入组时采集5 mL静脉血。血样采集后于1 h内以3 000 r/min离心10 min分离血清。离心后，研究人员在分装前对血清外观进行质量评估，剔除存在明显溶血或脂血的异常样本。其余血清样本随后分装入1.5 mL冻存管，并立即置于-80 ℃保存。产妇基本信息和新生儿数据由医院病历系统提取，包括年龄、孕前BMI、教育水平、分娩方式、胎次、体重及妊娠期糖尿病情况。分娩后由产科护士测量并记录新生儿出生体重、身长、头围及孕周。

### 1.3 仪器与试剂

ExionLC^TM^ AC 液相色谱-6500 QTRAP三重四极杆质谱（美国AB SCIEX 公司）；Architect i2000SR 分析仪（美国Abbott公司）；Maglumi 800分析仪（中国深圳新产业医学工程股份有限公司）；超纯水仪（美国Millipore公司）。

5%牛血清白蛋白（bovine serum albumin，BSA）溶液（美国Sigma-Aldrich公司）；睾酮、DHEAS与皮质醇的CLIA试剂盒（美国Abbott 公司）；17-OHP CLIA试剂盒（中国深圳新产业医学工程股份有限公司）；乙腈、甲醇、乙酸乙酯、甲酸及氨水（色谱纯，中国安谱实验科技股份有限公司）；皮质醇、DHEAS、睾酮和17-OHP的标准物质和同位素内标（中国曼哈格生物科技有限公司）。

### 1.4 LC-MS/MS样本前处理

本研究采用基于稳定同位素内标的LC-MS/MS定量分析法对4种类固醇激素（皮质醇、DHEAS、睾酮和17-OHP）进行检测，所使用的内标分别为皮质醇-d4、DHEAS-d6、睾酮-d5和17-OHP-d8。首先，将200 μL血清等分试样在室温下解冻，紧接着加入300 μL含有内标（10 ng/mL）的乙腈溶液。将混合液涡旋振荡1 min后，再添加500 μL乙酸乙酯。随后，以2 000 r/min的转速涡旋5 min，之后以12 000 r/min的转速离心5 min。完成上述操作后，把600 μL上清液转移至96孔板中。接下来，使用氮吹仪以氮气吹干转移后的上清液提取物，浓缩完毕后，用100 μL甲醇-水（1∶1，体积比）稀释剂重新溶解提取物。在进行分析之前，每个样品需以800 r/min的转速涡旋振荡5 min。

### 1.5 分析条件

色谱条件：皮质醇、睾酮和17-OHP的检测采用ESI^+^模式，使用Thermo Accucore PFP色谱柱（100 mm×2.1 mm，2.6 μm，美国赛默飞公司）。流动相由0.1%甲酸水溶液（A）和0.1%甲酸甲醇溶液（B）组成，流速设定为0.45 mL/min，梯度洗脱程序如下：0~3.20 min，39%B~42%B；3.20~7.50 min，42%B~90%B；7.50~7.51 min，90%B~100%B；7.51~8.50 min，100%B；8.51~10.00 min，100%B~39%B。DHEAS的检测采用ESI^–^模式，使用Phenomenex Titank C18色谱柱（100 mm×2.1 mm，3 μm，美国菲罗门公司），流动相由0.04%氨水溶液（A）和乙腈（B）组成，流速设定为0.4 mL/min，梯度洗脱程序如下：0~1.50 min， 35%B~60%B； 1.50~3.00 min， 60%B~95%B； 3.00~4.50 min，95%B~35%B。

质谱条件：ESI源。ESI^+^模式下，电喷雾电压设为+5 500 V，离子源温度为600 ℃，幕气压力为241 kPa，碰撞气设置为中等，GS1（喷雾气）和GS2（干燥气）均为高纯氮气，压力均为345 kPa。ESI^–^模式下，电喷雾电压设为-4 500 V，离子源温度为600 ℃，幕气压力为138 kPa，碰撞气设置为高强度，GS1和GS2压力分别为345 kPa和414 kPa。其他质谱参数见表1。

**表1:** 类固醇激素定量分析的优化质谱参数

Hormone	RT/min	Quantitative analysis					Qualitative analysis			
		Ion pair （*m*/*z*）	DP/V	CE/eV	Dwell time/ms		Ion pair （*m*/*z*）	DP/V	CE/eV	Dwell time/ms
Cortisol	4.59	363.3/121.2	100	25	25		363.3/155.1	80	25	25
Cortisol-d4	4.58	367.1/121.2	100	34	25		/			
DHEAS	0.80	367.1/97.0	-120	-30	25		367.1/80.0	-120	-80	25
DHEAS-d6	0.80	373.3/98.0	-150	-38	25		/			
Testosterone	6.61	289.2/97.2	90	28	25		289.2/109.0	90	33	25
Testosterone-d5	6.59	294.1/100.3	40	30	25		/			
17-OHP	6.70	331.2/97.2	80	30	25		331.2/109.2	80	33	25
17-OHP-d8	6.40	339.1/100.1	80	35	25		/			

RT： retention time； DP： declustering potential； CE： collision energy； DHEAS： dehydroepiandrosterone sulfate； 17-OHP： 17*α*-hydroxyprogesterone.

### 1.6 CLIA检测

皮质醇、睾酮和DHEAS采用美国雅培公司Architec i2000R分析仪及其配套试剂盒检测，17-OHP采用深圳新产业公司Maglumi 800仪器及其试剂盒进行测定。所有样本经3 500 r/min离心8 min后分离血清，并按说明书进行预处理与分析。质量控制方面，所有批次均使用高、低浓度质控样本进行内部质控，并进行重复测量。

### 1.7 统计学分析

本研究对参与者的一般人口学特征进行了描述性统计分析。连续变量以“均值±标准差（standard deviation，SD）”或“中位数（四分位距，interquartile range，IQR）”表示，分类变量则以频数和百分比（*n*，%）呈现。一致性分析采用配对设计，对同一样本分别通过LC-MS/MS与CLIA测定4种激素水平。在进行方法间一致性评估前，首先采用Shapiro-Wilk检验对4种激素的原始浓度数据进行正态性检验。若数据满足正态分布假设，则采用配对*t*检验评估两种方法之间的测量差异；若不满足，则采用Wilcoxon符号秩检验。两种方法中类固醇激素的分布采用配对箱式图进行展示。为进一步考察两种方法间的一致性，采用Bland-Altman分析评估各个浓度水平下测量差异的分布及一致性限，明确CLIA与LC-MS/MS在个体水平的偏差趋势与临床可接受范围。此外，采用Passing-Bablok回归法以非参数方式稳健检验两方法之间的系统性偏倚（截距显著偏离0）和比例性偏倚（斜率显著偏离1），以判断其可替代性。所有统计分析均使用R语言（版本4.3.1），其中Bland-Altman分析使用“BlandAltmanLeh”包，Passing-Bablok回归使用“mcr”包。统计显著性水平设定为*P*<0.05（双侧检验）。

## 2 结果与讨论

### 2.1 MS条件优化

按1.4节的样本前处理与1.5节色谱分离条件建立全扫描方法，根据化合物信息确定母离子后运行子离子扫描，选择丰度最高的两对离子对作为目标化合物的定量离子对和定性离子对，并确定每对离子对的最佳碰撞能量。所有MRM离子对的驻留时间统一设置为25 ms，确保采集点密度满足定量分析需求。优化后4种类固醇激素与各内标的质谱分析参数见表1。

### 2.2 线性范围、检出限与定量限

以5% BSA溶液作为模拟基质，向其中加入不同浓度的混合标准溶液，配制系列质量浓度的标准溶液。其中，皮质醇的系列质量浓度为5、10、25、50、250、500、1 000 ng/mL；DHEAS为20、40、100、200、1 000、2 000、4 000 ng/mL；睾酮为0.05、0.1、0.25、0.5、2.5、5、10 ng/mL；17-OHP为0.2、0.4、1、2、10、20、40 ng/mL。采用内标法进行定量分析，以目标化合物与相应内标的定量离子峰面积之比为纵坐标（*Y*），目标化合物的质量浓度为横坐标（*X*，ng/mL），绘制标准曲线。采用1.5节分析条件，各目标化合物的线性范围、回归方程、决定系数（*R*²）、检出限（LOD）和定量限（LOQ）见表2。4种类固醇激素在各自的线性范围内均呈良好的线性关系，*R*²均≥0.999。实验中，以信噪比（signal-to-noise ratio，*S/N*）为3时对应的质量浓度定义为LOD，以*S/N*为10时对应的质量浓度定义为LOQ。经测定，4种激素的LOD范围为0.002~0.042 ng/mL，LOQ范围为0.008~0.137 ng/mL。

**表2:** 类固醇激素的回归方程、线性范围、LOD与LOQ

Hormone	Regression equation	*R*²	Linear range/ （ng/mL）	LOD	LOQ
Cortisol	*Y*=0.042*X*-0.041	0.999	5-1000	0.008	0.025
DHEAS	*Y*=0.956*X*+5.463	0.999	20-4000	0.042	0.137
Testosterone	*Y*=0.94*X*+0.046	0.999	0.05-10	0.002	0.008
17-OHP	*Y*=1.026*X*-0.030	0.999	0.2-40	0.025	0.083

*R* ^2^： coefficient of determination； LOD： limit of detection， ng/mL； LOQ： limit of quantification， ng/mL. Standard curves were constructed by plotting the peak area ratio of each analyte to its corresponding internal standard （*Y*） against the analyte mass concentration （*X*， ng/mL）.

### 2.3 方法的准确度与精密度

采用5% BSA溶液作为模拟基质进行加标回收试验，分别加入不同浓度的类固醇激素混合标准溶液，配制成低、中、高3个浓度等级的加标样本，每个浓度设6个平行样。计算加标回收率及相对标准偏差（RSD）。样本检测过程中同步分析空白5% BSA溶液和超纯水基质，用于监测仪器性能稳定性并控制潜在背景干扰。结果显示，本研究建立的LC-MS/MS方法具有良好的准确性与精密度，能够满足孕期类固醇激素的定量需求（见表3）。

**表3:** 类固醇激素在3个水平下的加标回收率和精密度（*n*=6）

Hormone	Low level				Medium level				High level		
	Added/（ng/mL）	Rec./%	RSD/%		Added/（ng/mL）	Rec./%	RSD/%		Added/（ng/mL）	Rec./%	RSD/%
Cortisol	15	108.3	5.6		75	110.9	9.0		375	92.1	5.9
DHEAS	150	110.2	3.2		750	104.6	5.1		3750	96.5	5.9
Testosterone	0.15	97.9	5.7		0.75	101.0	6.0		3.75	100.7	6.3
17-OHP	0.6	99.8	4.8		3	100.8	5.0		15	100.1	3.6

Rec.： recovery； RSD：relative standard deviation.

对于孕期人群而言，体内激素水平受孕期生理变化的显著影响，部分关键激素的浓度较低，对检测方法的灵敏度提出了更高要求。Yuan等^［10］^采用与本研究相同的同位素内标和液-液提取流程，其方法中睾酮的LOQ为0.01 ng/mL，质量控制样本的RSD范围为6.4%~10.3%。相比之下，本研究在保证检测性能的基础上，通过简化前处理流程并省略衍生化步骤，不仅将睾酮的LOQ降低至0.008 ng/mL，还有效减少了操作复杂性和批间差异。此外，与Debeljak等^［11］^开发的方法相比，本研究在多项激素的定量限上表现出更高的灵敏度。例如，Debeljak等^［11］^开发的方法中皮质醇和DHEAS的LOQ分别为1.45 ng/mL和24.32 ng/mL，明显高于本研究所达到的0.025 ng/mL和0.137 ng/mL。上述比较进一步验证了本研究方法在微量类固醇激素检测方面的优势，为孕期关键激素的精确监测提供了更为可靠的技术支撑。

### 2.4 一致性分析中人群的基本特征

本研究共纳入94名孕妇进行4种激素在LC-MS/MS与CLIA方法的一致性分析。如表4所示，平均年龄为（31.15±4.14）岁，采样时孕周为（14.25±1.35）周，孕前BMI为（21.70±2.98） kg/m²。初产妇占53.2%，经阴道分娩者占59.6%，妊娠期糖尿病发生率为19.1%，男婴比例为46.8%。

**表4:** 研究人群的基本特征

Characteristic	Mean±SD or *n* （%）
Maternal age/year	31.15±4.14
Gestational age at sampling/week	14.25±1.35
Pre-pregnancy BMI/（kg/m^2^）	21.70±2.98
Pre-pregnancy BMI categories	
Underweight （<18.5 kg/m^2^）	8 （8.5）
Normal-weight （18.5-23.9 kg/m^2^）	74 （78.7）
Overweight （>23.9 kg/m^2^）	12 （12.8）
Nulliparous	50 （53.2）
Vaginal delivery	56 （59.6）
Gestational diabetes	18 （19.1）
Male infant	44 （46.8）

SD： standard deviation； BMI： body mass index.

### 2.5 配对分析结果

表5和图1展示了LC-MS/MS与CLIA两种方法对皮质醇、DHEAS、睾酮和17-OHP检测结果的配对箱式图。从分布总体来看，两种方法在上述4种激素的测定结果中均存在显著差异。具体而言，LC-MS/MS测得的皮质醇和DHEAS水平均显著低于CLIA（*P*<0.001），而睾酮和17-OHP则显著高于CLIA（*P*<0.001）。箱式图中灰色连线表示同一样本在两种检测方法下的配对结果，反映出一致的系统性偏倚趋势。

**表5:** 血清中4种类固醇激素的LC-MS/MS与CLIA定量检测结果对比

Parameter	Cortisol			DHEAS			Testosterone			17-OHP	
	LC-MS/MS	CLIA		LC-MS/MS	CLIA		LC-MS/MS	CLIA		LC-MS/MS	CLIA
Mean/（ng/mL）	64.31	120.60		532.44	1612.10		1.10	0.88		2.00	1.07
SD/（ng/mL）	23.32	39.62		253.02	656.34		0.85	0.64		0.89	0.50
Minimum/（ng/mL）	16.40	35.00		85.20	278.00		0.20	0.20		0.87	0.37
25th/（ng/mL）	47.98	94.00		329.25	1138.00		0.65	0.54		1.48	0.69
50th/（ng/mL）	59.55	115.00		535.50	1636.50		0.87	0.71		1.78	0.93
75th/（ng/mL）	77.88	142.00		702.25	2005.00		1.21	0.94		2.28	1.36
Maximum/（ng/mL）	127.00	225.00		1380.00	3536.00		5.40	4.21		6.27	2.99
Difference/（ng/mL）	-56.29			-1079.68			0.22			0.89	
%Difference	-46.52%			-67.49%			24.47%			93.82%	
*t*-value	26.36			24.13			-7.98			-13.44	
*P*-value	<0.001			<0.001			<0.001			<0.001	

CLIA： chemiluminescence immunoassay； Detection of 17-OHP was only performed in 72 samples due to the restriction of sample volume. Difference represents LC-MS/MS-CLIA； %Difference represents （LC-MS/MS-CLIA）/CLIA×100%.

**图1:**
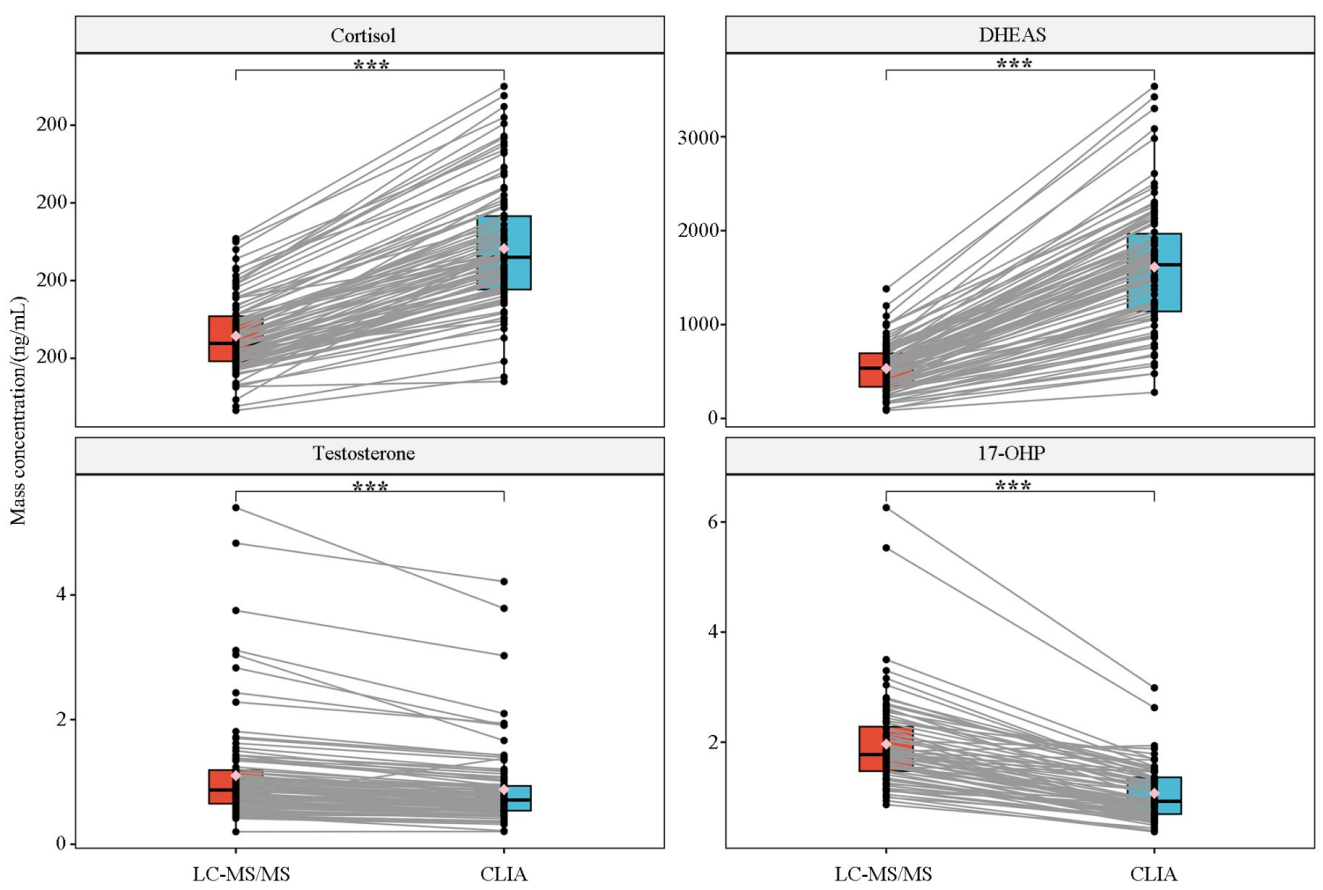
4种类固醇激素在LC-MS/MS与CLIA检测方法间的配对箱式图

### 2.6 Bland-Altman分析

图2展示了采用Bland-Altman方法对LC-MS/MS与CLIA在测定皮质醇、DHEAS、睾酮和17-OHP浓度结果的一致性分析图。结果显示，两种方法检测皮质醇与DHEAS的质量浓度差值分别为-56.29 ng/mL和-1 079.68 ng/mL，且一致性上下限范围较宽（皮质醇：-98.87~-15.71 ng/mL；DHEAS：-1 930.11~-229.25 ng/mL），表明LC-MS/MS测定值显著低于CLIA，且二者在高浓度范围内差值逐渐加大，呈现明显的负向比例偏倚。相反，两种方法检测睾酮和17-OHP的浓度差值分别为0.22 ng/mL和0.89 ng/mL，提示LC-MS/MS测定值整体高于CLIA，且一致性上下限范围相对较窄（睾酮：-0.31~0.76 ng/mL；17-OHP：-0.21~2.00 ng/mL），显示两方法间具备较好的数值一致性。然而，图中散点分布提示睾酮与17-OHP同样存在一定程度的比例偏倚，即随着浓度升高，LC-MS/MS与CLIA之间的浓度差值呈相应变化。综上所述，4种激素均存在不同程度的系统性偏倚，其中皮质醇与DHEAS偏倚程度显著，表明二者在LC-MS/MS与CLIA间存在不可忽略的测量差异，需在临床解释时加以注意。

**图2:**
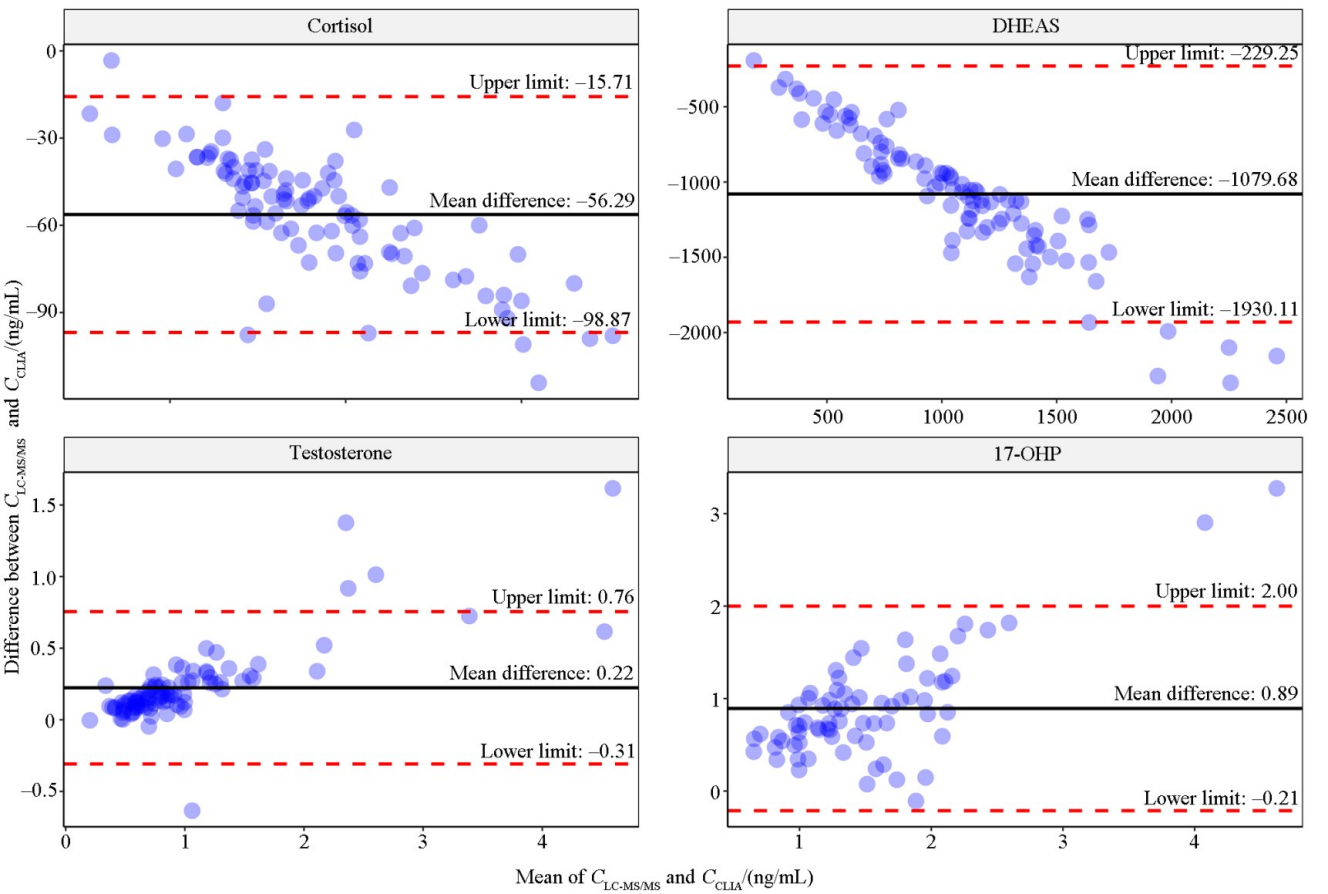
LC-MS/MS与CLIA法检测4种激素的 Bland-Altman 图

### 2.7 Passing-Bablok回归分析

表6与图3展示了LC-MS/MS与CLIA两种方法在皮质醇、DHEAS、睾酮和17-OHP中的Passing-Bablok回归分析结果。Pearson相关分析结果显示，两种方法在所有激素中均具有极强的正相关（*P*<0.001），相关系数*r*为0.819~0.974，其中睾酮结果的相关性最高（*r*=0.974）。皮质醇的一致性回归方程中斜率的置信区间不包含1，提示存在明显的比例性偏倚。本研究发现，LC-MS/MS测得的皮质醇浓度显著低于CLIA，且两者之间的差值随浓度升高而加大，表明两种方法在皮质醇测定中的一致性较差。这一观察与既往文献报道一致。Bae等^［12］^发现，免疫法测得的皮质醇浓度约为LC-MS/MS的2.39倍，其主要差异源于检测系统间标准化方式的不同。此外，当免疫法测得的皮质醇质量浓度低于1.81 ng/mL时，因可的松产生的交叉反应影响显著增强，进一步降低了两种方法间的一致性。Chafkin等^［8］^也报道了类似的比例性偏倚，指出CLIA法在整个浓度范围内普遍高估皮质醇浓度。Debeljak等^［11］^进一步证实，无论在低浓度还是高浓度区间，免疫分析法均表现出系统性的比例性偏倚，提示该问题具有一定的普遍性和稳定性。

**表6:** LC-MS/MS与CLIA法检测4种激素的Passing-Bablok回归分析结果

Hormone	Regression equation	Intercept （95% CI）	Slope （95% CI）	Bias direction
Cortisol	*C*_LC-MS/MS_=-3.58+0.57*C*_CLIA_	-3.58 （-8.17， 0.84）	0.57 （0.53， 0.61）^b^	LC-MS/MS<CLIA
DHEAS	*C*_LC-MS/MS_=-59.77+0.38*C*_CLIA_	-59.77 （-109.97， -19.58）^a^	0.38 （0.35， 0.41）^b^	LC-MS/MS<CLIA
Testosterone	*C*_LC-MS/MS_=-0.05+1.30*C*_CLIA_	-0.05 （-0.11， -0.005）^ a^	1.30 （1.23， 1.39）^b^	LC-MS/MS>CLIA
17-OHP	*C*_LC-MS/MS_=0.10+1.75*C*_CLIA_	0.10 （-0.14， 0.40）	1.75 （1.39， 2.04）^b^	LC-MS/MS>CLIA

a. constant bias； b. proportional bias. CI： confidence interval.

**图3:**
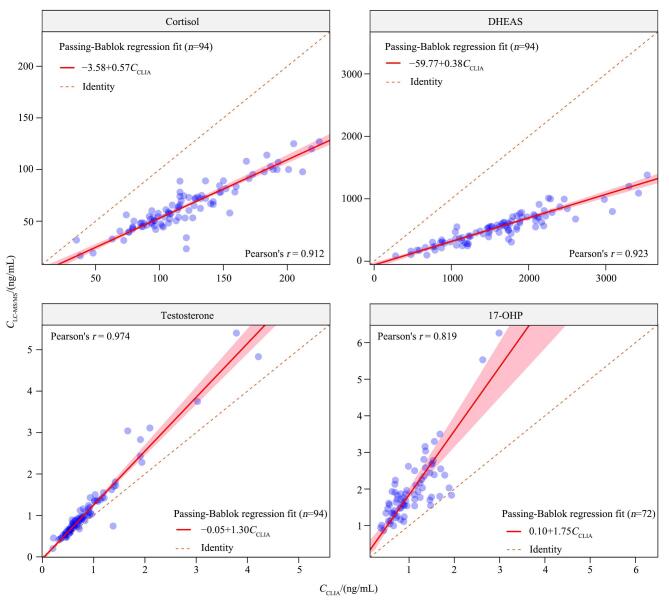
LC-MS/MS与CLIA法检测4种激素的Passing-Bablok回归分析

DHEAS的一致性回归方程中截距与斜率的置信区间分别不包含0和1，表明两种方法在DHEAS测定中同时存在固定性和比例性偏倚，且CLIA浓度显著高于LC-MS/MS。本研究中，DHEAS的测定差异尤为明显，最大质量浓度差值达1 079.68 ng/mL，提示两种检测方法在该激素的定量结果上存在显著的系统性差异，需在临床解释中谨慎处理。研究表明，孕期孕烯醇酮硫酸盐水平显著升高，其结构与DHEAS高度相似，可能引发免疫分析抗体的交叉反应，导致CLIA测定值偏高，这一机制已被多项研究所证实^［6，11］^。此外，夏曦等^［13］^研究指出，在多囊卵巢综合征女性中，LC-MS/MS测得的DHEAS 在诊断效能与灵敏度上优于CLIA，进一步支持了LC-MS/MS在低浓度、临界值判断中的临床优势。

睾酮作为母体主要的雄激素，尽管在妊娠期血清中的浓度相对较低，但其在维持母体内分泌稳态、支持胎盘功能及介导胎儿性别分化等方面具有关键生理作用，因此其在孕期的精准检测具有重要临床意义^［1］^。本研究发现，LC-MS/MS与CLIA两种方法在孕期女性睾酮测定中具有较好的一致性，但仍存在一定固定性和比例性偏倚，尤其在高浓度时差异扩大，提示两种方法虽具可比性但结果不可直接等同。Shi等^［14］^的研究同样发现，CLIA测得的睾酮值平均比LC-MS/MS低约20%，且质量浓度低于1 ng/mL时两法相关性一般。McCartney等^［15］^对青春期女性的研究同样显示两种方法结果之间具有良好的相关性，但一致性分析仍需关注。有报道指出不同免疫平台之间睾酮结果差异巨大，和LC-MS/MS相比可能正偏倚高达+137%。已有多项研究质疑了免疫法在低浓度检测中的可靠性。例如，Taieb等^［16］^认为现有免疫法不适用于儿童及女性等低浓度人群，另一项研究^［17］^亦显示LC-MS/MS对于检测青春期早期睾酮变化更敏感，能较免疫法提前约1.5年发现青春期睾酮变化。

17-OHP常用于先天性肾上腺皮质增生症筛查，尽管在妊娠期并非常规检测项目，其检测在评估女性高雄激素血症、多囊卵巢综合征、母体内分泌状态等方面，以及识别高风险孕妇个体具有较高的临床参考价值。本研究发现，两种方法在17-OHP测定中存在显著比例性偏倚，LC-MS/MS测值普遍高于CLIA，且差异随浓度升高而扩大，低浓度区域表现出系统性低估，需谨慎解读。本研究结论与Ambroziak等^［18］^在高雄激素血症女性中的研究具有一定可比性，发现两种方法之间存在显著差异，且偏差在低浓度区间更为明显，表现为系统性和比例性偏倚并存。既往报道还指出，LC-MS/MS测定值在部分研究中低于CLIA，因此建议基于质谱法建立新的诊断阈值^［11］^。此外，不同免疫平台间17-OHP结果差异巨大，偏倚最高可达+137%，可能导致假阳性误诊^［19］^。目前尚无孕妇人群中17-OHP水平在两种方法间比较的相关研究，因此本研究结论仍需进一步验证。

在类固醇激素检测中，免疫法因抗体交叉反应、基质干扰及灵敏度限制，常被认为倾向高估实际的激素水平，尤其在低浓度样本中更为明显。然而，本研究中睾酮和17-OHP的CLIA测定值却显著低于LC-MS/MS，与文献报道不一致。这种差异可能与孕期特有的生理状态和血浆基质的显著变化密切相关，提示在该人群中，传统免疫学方法可能面临更复杂的干扰因素。首先，孕期激素结合蛋白（如性激素结合球蛋白和皮质醇结合球蛋白）水平显著升高，游离激素比例下降，CLIA对结合型激素的识别能力有限，可能导致信号抑制和浓度低估^［14，20］^。其次，孕期血容量增加、脂蛋白水平升高、黏稠度变化等均可能干扰免疫分析的酶促或发光反应，造成非分析性偏差^［21］^。此外，孕妇胎盘和肾上腺分泌大量激素前体和代谢物，如硫酸孕烯醇酮、16-羟基类固醇等，可能与测定17-OHP或DHEAS的抗体发生交叉反应^［6，11］^。对于17-OHP而言，孕酮水平的剧增也可能通过微弱交叉反应或基质影响干扰免疫检测^［21］^。睾酮和17-OHP分子结构较小且相似，当抗体特异性不足时，识别效率下降或非特异结合减少，亦可能导致结果偏低。最后，CLIA平台间在抗体特异性、校准标准等方面存在差异，不同品牌间的一致性差表现尤为显著，尤其在低浓度激素测定中可能影响结果可比性。这些因素共同导致本研究中免疫法在部分激素的测定中出现系统性低估现象。

## 3 结论

本研究建立了一种可同时测定孕妇血清中皮质醇、DHEAS、睾酮及17-OHP的LC-MS/MS方法，并系统评估了该方法与CLIA的一致性。结果显示，两种方法在激素检测上存在显著差异，表现为不同程度的系统性偏倚，提示其结果不可直接互换。尤其在孕期激素水平波动大、浓度偏低的背景下，检测方法的选择将显著影响临床解读。本研究存在一定的局限性。首先，回收率验证采用5% BSA模拟基质，与真实孕妇血清仍存差异。其次，样本多来自妊娠早期，结论对中晚期的适用性需进一步验证。最后，CLIA结果基于单一平台，尚不能外推至其他系统。未来应结合大样本、多平台研究，涵盖全孕期及高风险人群，推动 LC-MS/MS 方法在产科激素监测中的标准化应用，并建立孕期特异的参考区间，以提升临床实用性和解读能力。
